# Effect of exercise on sleep quality in Parkinson’s disease: a mini review

**DOI:** 10.1186/s12883-024-03548-9

**Published:** 2024-01-30

**Authors:** M. Abdullah Shafiq, Jyotpal Singh, Zain A. Khan, J. Patrick Neary, Holly A. Bardutz

**Affiliations:** 1 College of Medicine, University of Saskatchewan Regina Campus, 1440 14 Ave, Regina, SK S4P 0W5 Canada; 2 Faculty of Kinesiology and Health Studies, University of Regina, 3737 Wascana Pkwy, Regina, SK S4S 0A2 Canada

**Keywords:** Parkinson’s Disease, Sleep, Exercise, Brain-derived neurotrophic factor, Neurotoxins

## Abstract

The growing incidence of Parkinson’s Disease (PD) is a major burden on the healthcare system. PD is caused by the degeneration of dopaminergic neurons and is known for its effects on motor function and sleep. Sleep is vital for maintaining proper homeostasis and clearing the brain of metabolic waste. Adequate time spent in each sleep stage can help maintain homeostatic function; however, patients with PD appear to exhibit sleep impairments. Although medications enhance the function of remaining dopaminergic neurons and reduce motor symptoms, their potential to improve sleep is still under question. Recently, research has shifted towards exercise protocols to help improve sleep in patients with PD. This review aims to provide an overview of how sleep is impaired in patients with PD, such as experiencing a reduction in time spent in slow-wave sleep, and how exercise can help restore normal sleep function. A PubMed search summarized the relevant research on the effects of aerobic and resistance exercise on sleep in patients with PD. Both high and low-intensity aerobic and resistance exercises, along with exercises related to balance and coordination, have been shown to improve some aspects of sleep. Neurochemically, sleeping leads to an increase in toxin clearance, including α-synuclein. Furthermore, exercise appears to enhance the concentration of brain-derived neurotrophic factors, which has preliminary evidence to suggest correlations to time spent in slow-wave sleep. More research is needed to further elucidate the physiological mechanism pertaining to sleep and exercise in patients with PD.

## Background

Parkinson’s Disease (PD), a progressive neurodegenerative disorder that impacts everyday life, is caused by the degeneration of dopaminergic neurons in the substantia nigra pars compacta, resulting in a significant dopamine deficiency [1]. The underlying cause explaining the loss of dopaminergic neurons remains enigmatic. However, emerging research suggests it may result from an interaction between genetic and environmental factors [1]. Although the symptoms vary depending on the individual, patients with PD exhibit motor and non-motor symptoms. Motor symptoms include tremors, rigidity, and postural imbalance, whereas, non-motor symptoms include poor sleep quality and quantity [2], and experiencing many sleep-wake disorders such as parasomnia [3], insomnia, sleep apnea, and restless leg syndrome [4].

Pharmaceutical interventions to strengthen dopaminergic functions and alleviate symptoms have been extensively researched as potential therapeutics for PD [5]. More recently, research has shifted towards observing changes in sleep parameters to assessing disease progression and the efficacy of the intervention, such as exercise. The purpose of this review is to summarize relevant research observing changes in overall sleep quality following exercise in patients with PD. We also provide a possible explanation due to the connection between exercise, brain-derived neurotrophic factors (BDNF), and various sleep parameters, including sleep quality, sleep disturbances, and sleep phases, in patients with PD.

## Methodology

### Search strategy

This literature review was narrative in nature and examines the influence of exercise on sleep quality in individuals with PD, while also associating a potential neuroprotective mechanism to explain the influence. A search of PUBMED and EMBASE (including Medline) was conducted to identify all studies investigating the effect of different exercise modalities on sleep in patients with PD until May 2023. Specifically, the search keywords were (Parkinson’s Disease OR Parkinson Disease) AND Exercise AND Sleep.

### Eligibility criteria

Our search resulted in 150 articles, and we examined the titles and abstracts to only include studies that discussed the influence of exercise on sleep in patients with PD. References from the included studies from the search were also hand-reviewed. We excluded study protocols, case studies, articles not focused on sleep changes due to an exercise protocol, and articles not in English.

### Data extraction and acquisition

We analyzed the studies and included their sample sizes, age range, stage of the participants’ disease, type of study, type of exercise completed, sleep assessment method, and results. To help describe a plausible neuroprotective mechanism, we also included studies that discussed the connection between BDNF and PD, specifically focusing on how BDNF is affected by exercise, and how this in turn impacts sleep in PD.

## Sleep and Parkinson’s disease

Sleep can be separated into phases of both non-rapid eye movement (NREM) and rapid eye movement (REM). NREM sleep consists of three progressively deeper stages of sleep, which include N1, N2 and slow-wave sleep (SWS) [6]. Conversely, REM sleep is thought to be related to emotional processing and is characterized by a reduction in muscular tone [7]. NREM sleep is associated with increases in slow-wave activity and delta-wave activity, whereas REM sleep is generally desynchronized and similar to an awakened state [8]. Although dreaming occurs in both the NREM and REM stages, the dreams during REM are more bizarre, emotional, and vivid [7].

It is well established that PD affects sleep [2, 9]. A systematic review and meta-analysis of polysomnography studies in patients with PD suggested that 90% of patients experienced sleep disorders, including insomnia, obstructive sleep apnea, and restless leg syndrome [2]. Whole sample analysis showed a decrease in REM, SWS percentage, and Mini-Mental State Examination, which is associated with a decrease in total sleep time and SWS [2].

While decreases in SWS have been linked to Alzheimer’s Disease due to the inability to decrease neuronal activity and potentially increase β-amyloid peptide concentration [10], larger-scale studies observing SWS and REM are limited in patients with PD. Schreiner et al. 2019 [11] suggested that increased slow-wave activity is associated with decreased progression of motor symptoms in patients with PD [11], whereas a 2012 review of case-control studies utilizing polysomnography on patients with PD suggested no substantial impairment in sleep stages and PD [12]. Therefore, whether improving SWS can potentially reduce PD symptoms, promote neurogenesis, and enhance neuroplasticity remains unknown.

### Clearance of toxins

Neurotoxicity in PD has been suggested, but concrete evidence is still lacking. Fultz et al. 2019 found a coupled relationship between neuronal activity, blood oxygenation, and cerebrospinal fluid (CSF) flow at low-frequency oscillations [13]. This suggests a communication between the neural and hemodynamic rhythms with CSF during SWS [13]. Interestingly, the majority of neurotoxin and metabolic removal by the CSF occurs during SWS via the glymphatic system [14]. Using radiolabeled β-amyloid in mice, researchers discovered that during sleep, the activity of the glymphatic system increased, leading to β-amyloids being removed twice as quickly compared to when the mice were awake [15]. Similar results were reported in humans after using positron emission tomography [16].

Furthermore, with the expansion of the interstitial space during SWS, the interstitial fluid and cerebrospinal fluid are able to properly interact and flush toxins, such as α-synuclein, via the perivascular pathways [14, 15]. α-Synuclein, a presynaptic neuronal protein linked to PD, is mainly located in pre-synaptic terminals and cell nuclei [15]. It provides a crucial role in regulating synaptic function, such as neurotransmitter release and enhancing synaptic plasticity [17]. Sleep disturbances compromise the function of the glymphatic system, resulting in an aggregation of α-synuclein proteins, which can accumulate and eventually lead to neurodegeneration [14]. One night of sleep deprivation can increase α-synuclein by up to 36% [16]. α-Synuclein aggregation is considered a primary cause of the varied symptoms and dysfunctions associated with PD, primarily due to α-synuclein forming toxic oligomers that result in cell death, along with the formation of Lewy bodies [18]. Accumulation of Lewy bodies can impair mitochondrial integrity and result in a neurotoxic and inflammatory environment that triggers neurodegeneration [19]. Therefore, by improving sleep, specifically SWS, in patients with PD and improving the function of the glymphatic system, the aggregation of α-synuclein decreases, possibly helping to reduce PD symptoms [14, 15, 20].

### Assessing sleep in patients with PD

Sleep is generally assessed through both subjective and objective methods. Subjective assessments include various questionnaires, the most common being the Pittsburgh Sleep Quality Index (PSQI), the Parkinson’s Disease Sleep Scale (PDSS), and the Parkinson’s Disease Sleep Scale 2 (PDSS-2). The PSQI is a tool that helps evaluate sleep quality and impairments. The PSQI consists of seven sections, which are scored between 0 and 3, and the sum of all the sections provides the global PSQI score [21]. Higher scores on the PSQI indicate low sleep quality [21], and patients with PD tend to have higher PSQI scores when compared to healthy controls, highlighting their lower sleep quality [22]. The PDSS is a visual analogue scale containing 15 questions associated with the various sleep issues experienced by patients with PD [23, 24]. The PDSS has been tested and has shown good test-retest reliability in patients with PD [23, 25]. Similarly, the PDSS-2 has 15 questions scored between 0 and 5, addressing 3 main domains, including PD symptoms, motor symptoms, and disturbed sleep [26, 27]. Similar to the PSQI, higher scores in the PDSS and the PDSS-2 indicate increased sleep abnormalities [26, 27].

There are two main objective techniques used to assess sleep in patients with PD: actigraphy and polysomnography. Actigraphy is a wrist accelerometer that helps understand the sleep-wake cycle through body movements and temperature [28]. Although actigraphy may overestimate sleep and underestimate wake time [29], it allows researchers to monitor sleep long-term [28, 30]. This helps healthcare providers diagnose sleep disorders and monitor treatment progress. In patients with PD, actigraphy assessments showed various impaired sleep parameters, including sleep efficiency [31], wake after sleep onset, and total sleep time as compared to controls [32]. Improved objective sleep measures are associated with improved morning mobility, implying a potential improvement in dopamine regulation [33].

Polysomnography (PSG) is the gold standard test [34] to measure sleep architecture and disorders [34], typically completed in a sleep clinic. PSG monitors sleep stages and records brain waves, blood oxygen levels, heart and breathing rate, and eye and leg movements during sleep [35]. Using PSG, Martinez-Ramirez et al. 2003 determined that about 57% of the participants with PD had obstructive sleep apnea, 49% had REM sleep behaviour disorder, 25% had periodic limb movement disorder, and 24% had awakenings/arousals [36]. Another study using overnight PSG determined patients with PD had more severe insomnia, shorter total sleep time, lower sleep efficiency, and increased REM latency when compared to controls [37]. By observing polysomnographic changes and global cognitive functioning, we can better understand the changes in REM in patients with PD, as well as better understand the associations with heart and breathing rates, brain wave activity, and motor abnormalities [9, 38].

## Improve sleep in patients with PD

Although sleep is negatively influenced by PD, exercising has been shown to reverse sleep impairments [2, 39]. Acute and regular exercise has been found to have small effects on total sleep time, SWS, and sleep onset latency [40]. A 12-week resistance training program showed a decrease in the PSQI global score in patients with PD, bringing the score closer to healthy controls [22]. Furthermore, based on the Mini-Sleep Questionnaire and the Pfeffer Questionnaire results, a six-month multimodal exercise program (which included resistance, balance, and aerobic fitness) also reduced various sleep disturbances, including insomnia and excessive daytime sleepiness, in patients with PD [41]. While many sleep studies are available to suggest a positive effect of exercise on sleep, the direct impact of acute and regular exercise on the various stages of sleep for patients with PD remains in question.

### Benefits of exercise on sleep

Exercise has been shown to be beneficial for physical functioning and presents a promising tool to improve and promote sleep in PD [42]. Exercise and physical activity are some of the best treatments for PD, and many studies now indicate how exercise may play a preventative role [43].

Patients in a multidisciplinary intensive rehabilitation treatment group consisting of a wide range of exercises demonstrated significant improvement in total Parkinson’s Disease Sleep Scale (PDSS) scores, including improvements in sleep quality [23]. A recent randomized, controlled trial showed improvements in sleep efficiency, time spent in SWS, total sleep time, and sleep architecture in patients with PD undergoing supervised resistance training exercise three times per week for sixteen weeks as compared to a group in sleep hygiene being given advice on sleep [44].

Exercises such as Qigong can be beneficial to aid individuals with limited movement [45]. Using the Parkinson’s Disease Sleep Scale 2 (PDSS-2), although both Qigong and sham-Qigong groups showed significant improvements in sleep quality and non-motor symptoms, the 12-week intervention of Qigong showed a more significant improvement [45]. Qigong and sham-Qigong performed similar exercises; however, the Qigong group also performed meditation and deep breathing with six healing sounds [45]. Nevertheless, both groups showed significant improvements, indicating that engaging in any form of physical activity can alleviate non-motor PD symptoms and improve sleep quality, regardless of the specific exercise type [45].

Tai Chi training three times per week has also been shown to decrease sleep disturbances and improve sleep quality as assessed by the PDSS in both individual and group training in patients with PD [46]. Baduanjin Qigong exercise has been shown to improve sleep symptoms on many of the parameters of the PDSS-2, including decreasing sleep disturbances and motor symptoms at night [47]. Finally, Qigong exercise has shown to improve motor symptoms at night in patients with PD, along with improvements in gait velocity [48].

Using accelerometers, an intervention of patients with PD completing 30 min of aerobic training followed by 30 min of resistance training showed an increase in sedentary activity during the overnight period [49]. This crucial finding suggests a reason for the improved sleep quality and daytime sleepiness [49]. The intervention may have induced a phenomenon known as sleep benefit, thereby helping the promotion of dopamine levels and dopaminergic function during sleep [49]. Sleep benefit is a phenomenon where individuals experience a decrease in various PD symptoms after waking up, such as having improved mobility. Typically, sleep benefit lasts for about 80 min after awakening but can last up to 5 h [50]. Research assessing the effects of resistance training in comparison to aerobic training on sleep in patients with PD can help to develop practical guidelines for neuro-rehabilitating patient populations.

Overall, exercise has been shown to increase sleep quality and sleep duration and decrease insomnia and daytime sleepiness. There are other modes of exercise used to assist and assess changes in sleep in neurodegenerative diseases not discussed in this review [42]; however, no direct mechanism is currently available to explain many of the physiological changes. An overview of studies which assess sleep following an exercise intervention in PD is included in Table 1.

**Table 1:** Studies of Parkinson’s disease and exercise with implications for sleep parameters

Author	Sample Size	Participant Age Range	Study Type	Disease Stage	Exercise Modality	Sleep Implication
Moon et al., 2020 [45]	17 (8 experimental group)	40–80 years of age	-Randomized Controlled Study - Double blind	Hoehn & Yahr stages 1–3	12-week Qigong exercise program performed twice per day at home (15–20 min) and once per week in a group session (45 min- 1 h)	Both sham and Qigong exercise improved sleep quality, and Actigraph variables (such as sleep efficiency) correlated with PD symptoms at night
Amara et al., 2020 [44]	55 (*n* = 27 experimental group)	≥ 45 years old	Randomized Controlled Study	Hoehn & Yahr stages 2–3	16-week resistance training and body-weight interval training at high intensity at 3 times per week	Improved sleep efficiency, total sleep time, wake after sleep onset, and slow-wave sleep; chronic exercise shows greater changes compared to acute.
Coe et al., 2018 [49]	65 (*n* = 29 experimental group)	Mean Age 67 years	Randomized Controlled Study -Single blind (assessors)	--	30 min of aerobic training followed by 30 min of resistance training	No impact on self-reported sleep, but increase in light activities overnight
Yang et al., 2017 [46]	36 (*n* = 19 in group training and 17 in individual training)	50–75 years old	Randomized Controlled Study -single blind (assessors)	Hoehn and Yahr stages 1–3	Tai Chi training 3 times a week for 13 weeks and daily home exercises	Both groups improved sleep quality (assessed by PDSS)
Silva-Batista et al., 2017 [22]	22 (11 in experimental group)	64–75 years old	Randomized Controlled Study - single blind (assessors)	Hoehn and Yahr stages 2–3	Resistance training program twice a week for 12 weeks	Resistance training resulted in improved PSQI sleep scores in patients with PD from pre-training to post-training
Xiao & Zhuang, 2016 [47]	100 PD (50 in experimental group)	55–80 years old	Randomized Controlled Study - single blind (assessors)	Hoehn and Yahr stages 1–3	Baduanjin Qiqong exercise for 6 months, completed at least once per day for at least 4 times per week	Improvements in sleep quality, and more specifically, decrease in motor symptoms at night and in disturbed sleep
Frazzitta et al., 2015 [23]	138 (89 in experimental group)	61–77 years old	Retrospective study	Hoehn and Yahr stages 2–3	Multidisciplinary intensive rehabilitation treatment: 4-week physical therapy that entailed three daily sessions, five days a week	Improvement in sleep quality as assessed by PDSS and UPDRS. Inverse correlation between levodopa and sleep scores
Wassom et al., 2015 [48]	7 PD	58–75 years old	Pre-Post Interventional trial	--	6-week Qigong exercise performed 2 times per day at home (15–20 min) and once per week as group (45–60 min)	Improved sleep quality, specifically, improved motor symptoms at night
Nascimento et al., 2014 [41]	64 (*n* = 17 PD in exercise and *n* = 17 PD in control; *n* = 30 in AD)	58–75 years old	Randomized Controlled Study - single blind (assessors)	Hoehn and Yahr stages 1–3	6-month exercise session (including resistance, aerobic and balance training), with three 1-hour sessions per week	Improved mini sleep questionnaire score in exercising group

PD: Parkinson’s Disease; PDSS: Parkinson’s Disease Sleep Scale; UPDRS: Unified Parkinson’s Disease Rating Scale; AD: Alzheimer’s Disease

## Parkinson’s disease and brain-derived neurotrophic factor (BDNF)

Brain-derived neurotrophic factor (BDNF) is a specific neurotrophic factor that provides developmental aid to neurons in the nervous system [51]. It stimulates the growth of new neurons and aids in the maturation and survival of existing neurons [51]. BDNF has been shown to protect neurons from various conditions, including cerebral ischemia and hypoglycemia [51]. PD is characterized by the loss of dopaminergic neurons, and studies have demonstrated that BDNF levels are lower in patients with PD [52]. Mogi and colleagues (1999) [52] found that in patients with PD, there was a significant decrease in BDNF levels in the nigrostriatal dopamine regions compared to healthy controls. Since BDNF confers neuroprotective properties, increasing and maintaining high levels of BDNF in patients with PD may be a potential therapeutic strategy. As per mouse models, activation of the BDNF signalling pathway could protect against loss of dopaminergic neurons in the substantia nigra [53]. This is also supported by a rat study which included a group with BDNF receptor blockade, causing the exhibition of the neuroprotective effects of BDNF to be lower compared to the control group with respect to the damage done to the dopaminergic system [54].

### Exercise and Brain-derived neurotrophic factor (BDNF)

A potential explanation underlying the improvement in sleep parameters due to exercise is the positive correlation between physical activity and BDNF levels in the brain. Sleiman et al. (2016) showed how exercising mice increased the production of β-hydroxybutyrate in the liver, which then travelled to the brain and inhibited histone deacetylase [55]. Histone deacetylases are a group of enzymes that remove acetyl groups from the amino acid lysine [56], and research demonstrates that histone deacetylase inhibitors, not only provide their own neuroprotective effects [56], but also help increase BDNF levels in the brain [55, 56]. These changes may help to better understand the neurological benefits associated with exercise, such as improving neurogenesis and neuroplasticity, and decreasing neuroinflammation and sleep impairments associated with PD.

Research has shown how BDNF may be a mediating molecule for the potential relationship between exercise and neurogenesis [57], a process that helps form new neurons in the brain [58]. Exercise and BDNF have been shown to promote growth and survival of neurons, thereby helping to recover various motor behaviours and decrease motor symptoms [59]. Hirsch et al. (2018) provides evidence for exercise increasing BDNF levels in the blood [59]; however, the underlying cause of exercise-induced BDNF remains elusive [60]. It is important to note that more research is still required to understand neurogenesis in patients with PD [58], as well as the potential impact of exercise-induce BDNF on neurogenesis.

Exercise also improves neuroplasticity [61], which is the brain’s ability to promote the formation and development of neural pathways. Neuroplasticity can be due to psychophysiological or environmental stimulation and results in functional or structural changes [61]. Kempermann et al. (2018) demonstrated that an increase in neurotrophins, such as BDNF, results in an increase in neuroplasticity [62]. Because of this finding, with the increases in BDNF following either acute or regular exercise [63], studies have found a potential correlation between exercise-induced BDNF and increases in neuroplasticity [59, 61, 62, 64]. For example, three cognitive rehabilitation sessions per week for one month in patients with PD involving paper and pencil exercises have shown improvements in serum BDNF levels and cognition [65]. The underlying mechanism explaining this correlation involves BDNF binding to its receptors, which results in the release of intracellular signals and subsequent phosphorylation of cAMP-response element-binding protein [60], a protein that regulates the expression of genes in dopaminergic neurons [66]. This phosphorylation increases the expression of tyrosine hydroxylase, an enzyme that helps convert the amino acid tyrosine into dopamine, resulting in exercise-dependent neuroplasticity [60].

Exercise has also been shown to aid in mitochondrial function and reduce neuroinflammation, which has been associated with PD [67]. Mitochondrial dysfunction in patients with PD can lead to apoptosis and oxidative stress due to the inhibition of complex I, causing an increase in reactive oxygen species and the destruction of dopaminergic neurons [68]. A mouse model showed that exercise can reduce the build-up of α-synuclein, along with increasing BDNF and heat shock protein (Hsp70) [69]. This is an important finding as it shows the therapeutic potential of exercise in patients with PD who have been shown to express low serum BDNF levels and increased α-synuclein levels, which has contributed to cognitive deficits [70, 71].

Treadmill exercise has also been shown to reduce impairments in striatal dopaminergic neurons and reduce oxidative damage in a rodent model of PD [72]. A mouse model of PD with moderate neurodegeneration showed that treadmill exercise reduced the loss of neuronal dopamine-producing cells and increased mitochondrial function and region-specific BDNF levels [73]. High-intensity exercises, which combine resistance training, aerobic training, and balance training, have been shown to increase mitochondrial complex activity [74], and lower-intensity exercises, such as walking, have been shown to increase maximum oxygen consumption [75]. Other chemical enhancements suggest increases in neural health due to aerobic treadmill exercises increasing levels of BDNF, neurogenesis, and the clearance of α-Synuclein [76]. Furthermore, moderate-intensity balance training has been shown to affect immune function in patients with PD, specifically by decreasing tumor necrosis factor-alpha [77].

Exercise modalities can be very important to assess since results show that high-intensity exercise combining both aerobic and resistance training increased BDNF levels more compared to moderate-intensity [78]. It has been suggested that increasing heart rate and oxygen demand can be beneficial when carefully monitored by a medical professional [79]. Both continuous and high-intensity cycle exercises have also been shown to increase serum BDNF in healthy humans; however, high-intensity exercises have been shown to result in greater BDNF concentrations [80].

### Sleep and BDNF

Sleep is associated with various neurochemical substances, including BDNF [81]. Although the mechanism remains unclear, altered BDNF levels have been shown to significantly influence sleep parameters [81]. A rat model found a strong positive correlation between BDNF in the pedunculopontine tegmentum and homeostatic drive towards REM sleep [82], suggesting that BDNF has a strong physiological mechanism to help increase REM sleep.

BDNF appears to be associated with sleep, as rat models have shown that microinjections of BDNF can result in increased slow-wave activity, and microinjections of the inhibitor of BDNF TrkB receptors resulted in decreased slow-wave activity [83]. Low BDNF levels were shown to be associated with low SWS and REM sleep in sleep-disordered patients [84].

Moreover, a study which induced microinjections of BDNF in awake rats at the prefrontal cortex showed significant increases in slow-wave activity during sleep, whereas the introduction of BDNF blockers resulted in decreased slow-wave activity [83]. This effect did not impact the duration of sleep and was reversible post-NREM [83]. Similarly, other research has found that BDNF levels during waking hours are directly correlated with SWS [85]. Therefore, it follows that if exercise can increase BDNF levels, it can increase slow-wave activity in patients with PD, resulting in better sleep. Nevertheless, the underlying mechanism(s) explaining the correlation between BDNF and SWS remains ambiguous and requires more research [85].

## Conclusion

Along with motor and non-motor impairments, research suggests patients with PD demonstrate various sleep disorders, including insomnia and obstructive sleep disorder. These issues contribute to the progression of motor symptoms and α-synuclein levels, resulting in homeostatic disruptions. Subjective questionnaires, polysomnography and actigraphy are used to assess sleep in patients with PD. However, a comprehensive approach needs to be implemented to observe the definitive REM and NREM sleep change mechanisms. Exercise has been shown to improve α-synuclein levels and sleep quality in individuals with PD. The largest sleep parameter improvement is shown through high-intensity interval group exercise. We hypothesize that the improved sleep parameters observed after exercise in patients with PD are due to the increased levels of BDNF; however, more human trials are required.

## Data Availability

Not Applicable.

## Abbreviations

PD: Parkinson’s Disease
BDNF: Brain-Derived Neurotrophic Factor
NREM: Non-rapid eye movement
REM: Rapid eye movement
SWS: Slow-wave sleep
CSF: Cerebrospinal fluid
PSG: Polysomnography
PSQI: Pittsburgh Sleep Quality Index
PDSS: Parkinson’s Disease Sleep Scale
UPDRS: Unified Parkinson’s Disease Rating Scale
